# Optimal Allocation of Water Resources and Eco-Compensation Mechanism Model Based on the Interval-Fuzzy Two-Stage Stochastic Programming Method for Tingjiang River

**DOI:** 10.3390/ijerph19010149

**Published:** 2021-12-23

**Authors:** Ning Hao, Peixuan Sun, Luze Yang, Yu Qiu, Yingzi Chen, Wenjin Zhao

**Affiliations:** 1 College of New Energy and Environment, Jilin University, Changchun 130012, China; haoning21@mails.jlu.edu.cn (N.H.); sunpx19@mails.jlu.edu.cn (P.S.); yanglz19@mails.jlu.edu.cn (L.Y.); 2 Northeast Asian Studies College, Jilin University, Changchun 130012, China; qiuyu075@163.com (Y.Q.); chenyz@jlu.edu.cn (Y.C.)

**Keywords:** Tingjiang river, “three lines, single project”, eco-compensation mechanism, water resources allocation, interval-fuzzy two-stage stochastic programming

## Abstract

In this work, based on the upper line of water resources utilization and the bottom line of water environmental quality of “Three Lines, Single Project”, a fuzzy optimization method was introduced into the Tingjiang River water resources optimal allocation and eco-compensation mechanism model, which is based on the interval two-stage (ITS) stochastic programming method. In addition, a Tingjiang River water resources allocation and eco-compensation mechanism model based on the interval fuzzy two-stage (IFTS) optimization method was also constructed. The objective functions of both models were to maximize the economic benefits of the Tingjiang River. The available water resources in the basin, the water environmental quality requirements, and regional development requirements were used as constraints, and under the five hydrological scenarios of extreme dryness, dryness, normal flow, abundance, and extreme abundance, the water resources allocation plan of various sectors (industry, municipal, agriculture, and ecology) in the Tingjiang River was optimized, and an eco-compensation mechanism was developed. In this work, the uncertainty of the maximum available water resources in each region and the whole basin was considered. If the maximum available water resources were too high, it would lead to a large waste of water resources, whereas if the maximum available water resources were too low, regional economic development would be limited. Therefore, the above two parameters were set as fuzzy parameters in the optimization model construction in this work. The simulation results from the IFTS model showed that the amount of water available in the river basin directly affects the water usage by various departments, thereby affecting the economic benefits of the river basin and the amount of eco-compensation paid by the downstream areas. The average economic benefit of the Tingjiang River after the optimization of the IFTS model simulation was [3868.51, 5748.99] × 10^8^ CNY, which is an increase of [1.67%, 51.9%] compared to the economic benefit of the basin announced by the government in 2018. Compared to the ITS model, the economic benefit interval of the five hydrological scenarios of extreme dryness, dryness, normal flow, abundance, and extreme abundance was reduced by 28.54%, 44.9%, 31.49%, 40.37%, and 36.43%, respectively, which can improve the economic benefits of the basin and provide more accurate decision-making schemes. In addition, the IFTS simulation showed that the eco-compensation quota paid by downstream Guangdong Province to upstream Fujian Province is [28,116.4, 30,738.6] × 10^4^ CNY, which is a reduction of [8461.404, 110,836] × 10^4^ CNY compared to the 2018 compensation scheme of the government. Compared to the ITS model, the range of eco-compensation values was observed to increase by 9.94%, 54.81%, 15.85%, 50.31%, and 82.90%, respectively, under the five hydrological scenarios, which reduces the burden of ecological expenditure downstream and provides a broader decision-making space for decision-makers and thus enables improved decision-making efficiency. At the same time, after the optimization of the IFTS model, the additional water consumption of the second stage of the Tingjiang River during the extremely dry year decreased by 62.11% compared to the results of the ITS model. The additional water consumption of the industrial sector decreased by 68.39%, the municipal sector decreased by 59.27%, and in the first phase of water resources allocation for 14 districts and counties in the Tingjiang River, industrial and municipal sectors are the main two sectors. After introducing the fuzzy method into the IFTS model, the difference in the water consumption by these two sectors in the basin under different hydrological scenarios can be alleviated, and the waste of water resources caused by too low water allocation or excessive water allocation can be avoided. The national and local (the downstream region) eco-compensation quotas can be indirectly reduced, and the risk of water resources allocation and eco-compensation decision-making in the basin can be effectively reduced.

## 1. Introduction

As a common and indispensable natural resource, water plays an important role in human social life, economic development, and other activities and is also the basis of survival for various organisms. Furthermore, water resource has become an important factor restricting China’s development [1,2]. China’s basin has numerous aspects, such as flows through the provinces and a large radius. Due to its large valley basin being located in regions with different economic development levels, leading industry differences (industry, tourism, service, etc.), and the related environmental standards, the question of large watershed ecological management existence has arisen, and this often leads to a variety of phenomena such as low utilization efficiency of water resources and lower reaches of the river basin [3,4,5].

In recent years, eco-compensation has attracted considerable attention from scholars. Eco-compensation is an emerging environmental management method for protection and sustainable utilization of the ecosystem that uses economic means to adjust, organize a reasonable configuration of the river basin water resources, reduce the inefficient utilization of water resources, arouse enthusiasm for ecological protection and coordination of river basin near each administrative unit of economic interest, and promote the sustainable development of the river basin as a whole. Therefore, it plays an important role in watershed ecological protection and has become a research hotspot in watershed management and watershed ecological environment protection [6,7,8]. Guan et al. [9] calculated the ecological damage compensation value and protection compensation value of Xiaohong River Basin in Zhumadian, China, in order to promote the quantification of eco-compensation and water environmental management of Xiaohong River Basin. They found that the eco-compensation focus of the Xiaohong River basin was pollution damage compensation. Taking the Jiuzhou River Basin in China as a typical case, Sun et al. [10] proposed a bottom-up evolutionary framework of the eco-compensation system and analyzed the behavioral tradeoffs between pig farming and the upstream and downstream areas in order to achieve the goal of quantifying short-term and long-term eco-compensation standards and maintain good water quality. Wang et al. [11] proposed a transboundary water quality and quantity eco-compensation standard model for eco-compensation problems of water-deficient rivers in northern China, represented by the Yongding River. They suggested coupling the water quality eco-compensation standard based on pollutant emission reduction with the water quantity eco-compensation standard based on the restoration cost method to coordinate the upstream and downstream interests and promote rational allocation of the eco-compensation quota. By studying the upstream and downstream Stackelberg game of watershed transboundary pollution control and eco-compensation, Chen et al. [12] found that increasing the proportion of eco-compensation can improve the chain reaction among the green investment cost, the pollutant transfer rate, and the eco-compensation rate, and effectively realize the decision-making of watershed pollution control. However, the above studies only consider the upper limit of water resources utilization and the requirements of regional economic development but ignore the change in the pollutant concentration in the basin caused by the uncertainty of the water quantity in the basin and the influence of the uncertain factors such as the type and quantity of pollutants in the process of economic development. This results in a certain deviation between the research studies and reality.

In the process of environmental management and planning of water resources and the establishment of an eco-compensation mechanism, there are inevitable uncertainties in the accuracy and quantification of factors such as the number of water resources, the pollutant discharge intensity, and the pollution control cost. This results in complex environmental management and planning of water resources [13]. At present, given the various uncertain factors in the environmental management and planning of water, interval programming, stochastic programming, and fuzzy programming are mainly adopted to carry out the optimization to reduce the decision-making risk during planning in actual situations [14,15]. Interval programming expresses the parameters and the influencing factors in the form of interval numbers and expresses the uncertainty in the parameters [16]. Based on the interval two-stage robust optimization model, He et al. [17] optimized the Yinma River water resource allocation system to improve the efficiency of water usage and reduce the system risks. Stochastic programming is based on the known probability distribution and uses the model to solve the programming problem with constraint parameters or objective functions as random variables [18], including the two-stage stochastic programming proposed by Dantzig and the opportunity-constrained programming proposed by Charnes [19,20]. Liao et al. [21] used a two-stage random water resource allocation model with interval parameters to adjust the recommended water replenishment scheme for the Momoge National Nature Reserve under different scenarios and constraints to restore the habitat of endangered water birds to the maximum extent. Yang et al. [22] took the interconnected river network project in the west of Jilin Province as the research object and analyzed the water resource allocation in the region under different hydrological scenarios based on the interval fuzzy bilateral chance-constrained stochastic programming model to improve the water diversion ratio of marshes and wetlands. However, in the optimization process of the interval two-stage model, the influence of the fuzzy uncertainty is often ignored, and the range of the interval value of the model results is large. This leads to the system having high failure risk and decision risk. Therefore, the fuzzy optimization method can be introduced to solve the above problems. This method uses a fuzzy set to provide the possibility of uncertain parameter value and balance the relationship between the target value and the decision risk. This has the advantage of not requiring the collection of a large amount of data for theoretical support [23,24,25]. Liao et al. [26] optimized the ecological water refill scheme in the Momoge National Nature Reserve based on the interval-parameter two-stage stochastic programming model (IPTSP) and found that the fuzzy uncertainty that existed in the optimization process could lead to greater decision-making risks. Thus, they introduced the fuzzy optimization method for further optimization. The simulation results thus obtained showed that the model optimized using this method can significantly reduce the decision-making risk.

Considering the abovementioned aspects, this work combines the upper line of water environment utilization and the bottom line of water environment quality with an eco-compensation mechanism. Based on the model of optimal allocation of water resources and eco-compensation mechanism in the Tingjiang River, incorporating the interval two-stage stochastic programming method (ITS model) developed by Qiu [27], the fuzzy optimization method has been introduced to construct the interval-fuzzy two-stage stochastic programming model (IFTS) of water resources allocation and eco-compensation mechanism in the Tingjiang River. The IFTS model shortens the interval range of the simulation results obtained using the ITS model and thus reduces the decision risk while providing efficient decision space. The IFTS model constructed in this work aims at the maximum economic benefit of the Tingjiang River and is constrained by the upper limit of industrial water consumption, the bottom limit of water environmental quality, and the requirements of regional development. At the same time, the upper limit of water resources utilization in each region and in the Tingjiang River was taken as the key constraint. The IFTS model simulation results can provide valuable suggestions for regional managers to allocate water resources, protect water environment quality in the Tingjiang River, and construct a reasonable eco-compensation mechanism in the upper and lower reaches of the Tingjiang River.

## 2. Case Study

### 2.1. Natural Situation and Optimum Adjustment Background of the Tingjiang River

**Figure 1:**
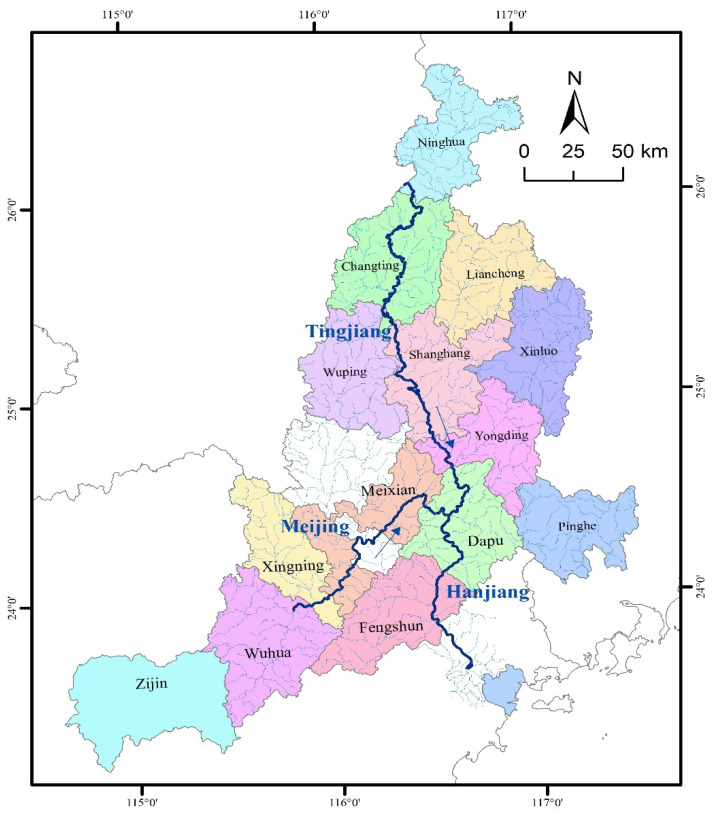
Natural situation of the Tingjiang River.

Figure 1 shows the main drainage system of the Tingjiang River. The Tingjiang River is the largest in western Fujian and contributes the most to the water flow from Fujian Province to Guangdong Province, with a total length of more than 300 km [28,29]. The Tingjiang River is one of the four areas with frequent rainfall in Fujian Province and Guangdong Province, and the average annual rainfall is as high as [1500, 2000] mm, which is unevenly distributed in space and time. Further, it gradually decreases from north to south and is mostly concentrated in May to July (accounting for 60% of the annual precipitation) [30]. At present, there are 32 water diversion projects of more than 66.7 ha in the Tingjiang River, and 19 small (primary) reservoirs, among which the total storage capacity of the Cotton Tan Reservoir is the largest. In the Water Resources Bulletin of Guangdong Province in 2018 (http://slt.gd.gov.cn/szygb2018/ accessed on 6 June 2002), its average comprehensive water consumption per capita was 374 m^3^, where the average water consumption per capita of urban residents was 69 m^3^, and that of rural residents was 47 m^3^.

There are 34 water function protection areas in five districts and counties of Longyan City, Fujian Province, in the upper reaches of the Tingjiang River, among which nine are drinking water source protection areas. In addition, adequate quantity and good quality of water are the interests of Guangdong Province in the lower reaches of the Tingjiang River [31,32]. Since 2016, the central and Guangdong and Fujian Provinces have invested capital of CNY 1.6 billion to protect the water environment and improve the quality of the river water. The 2017 compensation agreement section on water quality of II class, involving the Tingjiang River across the province, described the overall achieved agreement requirements and goals [33]. However, there is still the possibility that the water quality will deteriorate due to the increasing erosion of soil and water in the Tingjiang River basin, as well as the impact of livestock pollution and serious ecological restoration problems in the mines. Therefore, it is particularly important to optimize and adjust the existing water resources allocation scheme and eco-compensation mechanism in the Tingjiang River basin and promote the standardization and scientific processing of basin management.

### 2.2. Determination of the Research Objects and Uncertain Parameters in the Tingjiang River

Fourteen counties along the Tingjiang River basin were selected (Liancheng, Shanghang, Wuping, Xinluo, Yongding, Pinghe, and Changting counties in Fujian Province; Guangdong province: Zijin county, Dapu County, Fengshun county, Meixian county, Wuhua County, Xingning County, and Chenghai District) as the research object. Taking the number of allocable water resources, the upper limit of water resources utilization, the bottom line of water environmental quality, and the regional development requirements as the model constraints, and taking the maximum economic benefit of the basin as the objective function, the model of water resources allocation and eco-compensation mechanism in the Tingjiang River based on the IFTS optimization method was established. The model was used to predict the maximum economic benefit, eco-compensation quota, and water allocation scheme. The hydrological situation of the Tingjiang River is uncertain, and the runoff has fluctuated greatly from 1965 to 2012, presenting a declining up-and-down trend [30]. According to the information on the water resources in Fujian Province of many years (http://slt.fujian.gov.cn/ accessed on 1 June 2018), the Tingjiang River watershed hydrology situation can be subdivided into five types of hydrological situations, namely, extreme dryness, dryness, normal flow, abundance, and extreme abundance. The probability of these five scenarios was set as 0.1, 0.3, 0.15, 0.25, and 0.2, respectively. At the same time, the upper limit of regional water resources utilization and the maximum available water amount in the basin were taken as variables to construct the fuzzy functions to reduce the influence of uncertainty and make the decision-making space more extensive.

## 3. Model Formulation

### 3.1. Construction of the IFTS Model for the Tingjiang River

This paper took the Tingjiang River as the research object, realized the maximum economic benefit, and established a reasonable eco-compensation scheme by optimizing the utilization and allocation of water resources. The water supply in the Tingjiang River is affected by climate, rainfall, and other factors, and the two-stage stochastic method was adopted, which completely follows the randomness of water supply. All parameters were uncertain and fluctuated in the process of water resource allocation and eco-compensation model establishment. Considering the uncertainty of parameters, the interval programming method was introduced to set parameter values in a reasonable interval. There were fuzzy uncertainties in the maximum available water and departmental water consumption in the Tingjiang River. Therefore, the maximization of the economic benefit of the Tingjiang River was taken as the objective function. Using the model of water resources allocation and eco-compensation mechanism in the Tingjiang River, based on the interval two-stage (ITS) planning method, the upper limit of water resources utilization (the upper limit of industrial water consumption, the upper limit of regional water consumption, etc.), the bottom limit of water environment quality, and the development requirements of various regions in the basin were taken as the constraints. Taking the upper limit of water consumption of each department and the total water consumption of the basin as fuzzy variables, the water resource utilization of each district and county of the basin as the first stage, and the water shortage caused by insufficient water supply as the second stage, the randomness and uncertainty of parameters, such as the number of water resources available for allocation, and the pollutant discharge coefficient of the Tingjiang River were considered comprehensively. The model of optimal allocation of water resources and eco-compensation mechanism in the Tingjiang River was established by using the IFTS optimization method. Various water allocation schemes under different hydrological scenarios were optimized and adjusted to maximize the economic benefits of the basin, coordinate the economic interests of the upper and lower reaches of the Tingjiang River, improve the water environment quality of the basin, and promote sustainable development of the basin ecology. In the IFTS model, “+” indicates the maximum value of the parameter, whereas “−” indicates the minimum value. The optimization model of water resources allocation based on the IFTS optimization method is as follows:

Objective function:

(1) $$\max=t^{\pm }(0\leq t\leq 1)$$

where $t^{\pm }$ represents the fuzzy membership interval of the model.

Constraints:

(1) Benefit constraint:

(2) $$f_{1}^{\pm }-f_{2}^{\pm }-f_{3}^{\pm }-f_{4}^{\pm }-f_{5}^{\pm }\geq f^{\prime \prime }-(1-t^{\pm })*(f^{\prime \prime }-f^{\prime })$$

(3) $$f_{1}^{\pm }=\sum_{j=1}^{I}\sum_{k=1}^{3}\sum_{m=1}^{M}NB_{jkm}^{\pm }\cdot ISOPT_{jkm}$$

(4) $$f_{2}^{\pm }=\sum_{j=1}^{I}\sum_{k=1}^{K}\sum_{m=1}^{M}WR_{jk}^{\pm }\cdot ISOPT_{jkm}$$

(5) $$f_{3}^{\pm }=\sum_{j=1}^{J}\sum_{k=1}^{3}\sum_{m=1}^{M}\delta_{jkm}^{\pm }\cdot ISOPT_{jkm}\cdot STC_{jkm}^{\pm }$$

(6) $$f_{4}^{\pm }=\sum_{j=J_{{}_{1}}+1}^{J_{2}}\sum_{k=1}^{3}\sum_{m=1}^{M}WRD_{j}^{\pm }\cdot \eta \cdot \left|\lambda \right|$$

(7) $$f_{5}^{\pm }=\sum_{h=1}^{5}P_{h}\cdot PNB_{h}^{\pm }\cdot DIS_{h}^{\pm }$$

(2) Constraints on the development and utilization of water resources in the Tingjiang River:
  The number of water resources used by different industries in different areas within the basin should not exceed the water consumption stipulated by the state [18]:
  (8) $$\sum_{m=1}^{M}ISOPT_{jkm}-DIS_{jkmh}^{\pm }\leq IWUL_{jk}^{\pm };\forall j,k,h$$
  The total amount of water resources allocated to each region in the Tingjiang River should meet the upper limit of water resources in each region:
  (9) $$\sum_{k=1}^{K}\sum_{m=1}^{M}ISOPT_{jkm}-DIS_{jkmh}^{\pm }\geq RWUL_{j}^{+}-(1-t^{\pm })*(RWUL_{j}^{+}-RWUL_{j}^{-});\forall j,h$$
  According to the requirements of the “Three Lines, Single Project”, each region in the Tingjiang River must meet the regional ecological water consumption constraints when allocating water resources [18]:
  (10) $$\sum_{m=1}^{M}ISOPT_{jkm}-DIS_{jkmh}^{\pm }\geq ECS_{j}^{\pm };\forall j,h,k=4$$
  The total amount of water allocated to each region in the Tingjiang River should not exceed the maximum allowable utilization of the basin:
  (11) $$\sum_{j=1}^{J}\sum_{k=1}^{4}\sum_{m=1}^{M}ISOPT_{jkm}-DIS_{jkmh}^{\pm }\geq TAWR^{+}-(1-t^{\pm })*(TAWR^{+}-TAWR^{-}),\forall h$$

(3) Requirements for water quality limitation in the Tingjiang River:
  Limits on the discharge of various pollutants in the Tingjiang River [18]:
  (12) $$\sum_{k=1}^{2}\sum_{m=1}^{M}(ISOPT_{jkm}-DIS_{jkmh}^{\pm })\cdot \xi_{jkmr}^{\pm }\cdot \gamma_{jkmr}^{\pm }+(ISOPT_{jkm}-DIS_{jkmh}^{\pm })\cdot \xi_{j3mr}^{\pm }\leq AEP_{jr}^{\pm };\forall j,r,h$$

(4) Regional development requirements in the Tingjiang River [18]:

(13) $$\sum_{j=1}^{J_{i}}\sum_{m=1}^{M}PNB_{jkm}^{\pm }\cdot (ISOPT_{jkm}-DIS_{jkmh}^{\pm })\geq DSL_{ik}^{\pm };\forall k,i=1$$

(14) $$\sum_{j=J_{1}+1}^{J_{i}}\sum_{m=1}^{M}PNB_{jkm}^{\pm }\cdot (ISOPT_{jkm}-DIS_{jkmh}^{\pm })\geq DSL_{ik}^{\pm };\forall k,i=2$$

### 3.2. Model Solving

Since the objective function of the model is to maximize the economic benefits of the Tingjiang River, the upper bound sub-model was solved first. It can be expressed as follows:

Objective function:

(15) $$\max=t^{+}(0\leq t\leq 1)$$

Constraints:

(16) $$f_{1}^{+}-f_{2}^{-}-f_{3}^{-}-f_{4}^{-}-f_{5}^{-}\geq f^{\prime \prime }-(1-t^{+})*(f^{\prime \prime }-f^{\prime })$$

(17) $$f_{1}^{+}=\sum_{j=1}^{I}\sum_{k=1}^{3}\sum_{m=1}^{M}NB_{jkm}^{+}\cdot ISOPT_{jkm}$$

(18) $$f_{2}^{-}=\sum_{j=1}^{I}\sum_{k=1}^{K}\sum_{m=1}^{M}WR_{jk}^{-}\cdot (ISOPT_{jkm}-\sum_{h=1}^{H}P_{h}\cdot DIS_{jkmh}^{-})$$

(19) $$f_{3}^{-}=\sum_{j=1}^{J}\sum_{k=1}^{3}\sum_{m=1}^{M}\delta_{jkm}^{-}\cdot (ISOPT_{jkm}-\sum_{h=1}^{H}P_{h}\cdot DIS_{jkmh}^{-})\cdot STC_{jkm}^{-}$$

(20) $$f_{4}^{-}=\sum_{j=J_{1}+1}^{J_{2}}\sum_{k=1}^{3}\sum_{m=1}^{M}WRD_{j}^{-}\cdot (ISOPT_{jkm}-\sum_{h=1}^{H}P_{h}\cdot DIS_{jkmh}^{-})\cdot \eta \cdot \left|\lambda \right|$$

(21) $$f_{5}^{-}=\sum_{h=1}^{H}P_{h}\cdot PNB_{h}^{-}\cdot DIS_{h}^{-}$$

(22) $$\sum_{h=1}^{H}\sum_{m=1}^{M}ISOPT_{jkm}-DIS_{jkmh}^{-}\leq IWUL_{jk}^{+};\forall j,k$$

(23) $$\sum_{h=1}^{H}\sum_{k=1}^{K}\sum_{m=1}^{M}ISOPT_{jkm}-DIS_{jkmh}^{-}\geq RWUL_{j}^{+}-(1-t^{+})*(RWUL_{j}^{+}-RWUL_{j}^{-});\forall j$$

(24) $$\sum_{h=1}^{H}\sum_{m=1}^{M}ISOPT_{jkm}-DIS_{jkmh}^{-}\geq ECS_{j}^{+};\forall j,k=4$$

(25) $$\sum_{h=1}^{H}\sum_{j=1}^{J}\sum_{k=1}^{4}\sum_{m=1}^{M}ISOPT_{jkm}-DIS_{jkmh}^{-}\geq TAWR^{+}-(1-t^{+})*(TAWR^{+}-TAWR^{-})$$

(26) $$\sum_{h=1}^{H}\sum_{k=1}^{2}\sum_{m=1}^{M}(ISOPT_{jkm}-DIS_{jkmh}^{-})\cdot \xi_{jkmr}^{-}\cdot \gamma_{jkmr}^{-}+(ISOPT_{j3m}-DIS_{j3mh}^{-})\cdot \xi_{j3mr}^{-}\leq AEP_{jr}^{+};\forall j,r$$

(27) $$\sum_{h=1}^{H}\sum_{j=1}^{7}\sum_{m=1}^{M}NB_{jkm}^{+}\cdot (ISOPT_{jkm}-DIS_{jkmh}^{-})\geq DSL_{ik}^{+};\forall k.i=1$$

(28) $$\sum_{h=1}^{H}\sum_{j=7}^{14}\sum_{m=1}^{M}NB_{jkm}^{+}\cdot (ISOPT_{jkm}-DIS_{jkmh}^{-})\geq DSL_{ik}^{+};\forall k,i=2$$

(29) $$ISOPT_{jkm}=IS_{jkm}^{+}+KN_{jkm}\cdot (IS_{jkm}^{+}-IS_{jkm}^{-});0\leq KN_{jkm}\leq 1$$

$ISOPT_{jkm}$ was solved by the upper bound sub-model. The water resources allocation of each water consumption department under different hydrological scenarios in the first stage of the Tingjiang River was analyzed, and the water shortage of each water consumption department under different hydrological runoff scenarios, $DIS^{-}$, was calculated. According to the interactive algorithm, the value of $ISOPT_{jkm}$, obtained in the upper bound sub-model, was taken as the constraint condition to solve the lower bound sub-model. It is expressed as follows:

Objective function:

(30) $$\max=t^{-}(0\leq t\leq 1)$$

Constraints:

(31) $$f_{1}^{-}-f_{2}^{+}-f_{3}^{+}-f_{4}^{+}-f_{5}^{+}\geq f^{\prime \prime }-(1-t^{-})*(f^{\prime \prime }-f^{\prime })$$

(32) $$f_{1}^{-}=\sum_{j=1}^{I}\sum_{k=1}^{3}\sum_{m=1}^{M}NB_{jkm}^{-}\cdot ISOPT_{jkm}$$

(33) $$f_{2}^{+}=\sum_{j=1}^{J}\sum_{k=1}^{3}\sum_{m=1}^{M}WR_{jk}^{+}\cdot (ISOPT_{jkm}-\sum_{h=1}^{H}P_{h}\cdot DIS_{jkmh}^{+})$$

(34) $$f_{3}^{+}=\sum_{j=1}^{J}\sum_{k=1}^{3}\sum_{m=1}^{M}\delta_{jkm}^{+}\cdot (ISOPT_{jkm}-\sum_{h=1}^{H}P_{h}\cdot DIS_{jkmh}^{+})\cdot STC_{jkm}^{+}$$

(35) $$f_{4}^{+}=\sum_{j=J_{1}+1}^{J_{2}}\sum_{k=1}^{3}\sum_{m=1}^{M}WRD_{j}^{+}\cdot (ISOPT_{jkm}-\sum_{h=1}^{H}P_{h}\cdot DIS_{jkmh}^{+})\cdot \eta \cdot \left|\lambda \right|$$

(36) $$f_{5}^{+}=\sum_{h=1}^{5}P_{h}\cdot PNB_{h}^{+}\cdot DIS_{h}^{+}$$

(37) $$\sum_{h=1}^{H}\sum_{m=1}^{M}(ISOPT_{jkm}-DIS_{jkmh}^{+})\leq IWUL_{jk}^{-};\forall j,k$$

(38) $$\sum_{h=1}^{H}\sum_{k=1}^{K}\sum_{m=1}^{M}(ISOPT_{jkm}-DIS_{jkmh}^{+})\geq RWUL_{j}^{+}-(1-t^{-})*(RWUL_{j}^{+}-RWUL_{j}^{-});\forall j$$

(39) $$\sum_{h=1}^{H}\sum_{m=1}^{M}(ISOPT_{jkm}-DIS_{jkmh}^{+})\geq ECS_{j}^{-};\forall j,k=4$$

(40) $$\sum_{h=1}^{H}\sum_{j=1}^{J}\sum_{k=1}^{4}\sum_{m=1}^{M}\left(ISOPT_{jkm}-DIS_{jkmh}^{+})\geq TAWR^{+}-(1-t^{-})*(TAWR^{+}-TAWR^{-}\right)$$

(41) $$\sum_{h=1}^{H}\sum_{k=1}^{2}\sum_{m=1}^{M}(ISOPT_{jkm}-DIS_{jkmh}^{+})\cdot \xi_{jkmr}^{+}\cdot \gamma_{jkmr}^{+}+(ISOPT_{j3m}-DIS_{j3mh}^{+})\cdot \xi_{j3mr}^{+}\leq AEP_{jr}^{-};\forall j,r$$

(42) $$\sum_{j=1}^{J_{i}}\sum_{m=1}^{M}NB_{jkm}^{-}\cdot (ISOPT_{jkm}-DIS_{jkmh}^{+})\geq DSL_{ik}^{-};\forall k,i=1$$

(43) $$\sum_{j=J_{1}+1}^{J_{i}}\sum_{m=1}^{M}NB_{jkm}^{-}\cdot (ISOPT_{jkm}-DIS_{jkmh}^{+})\geq DSL_{ik}^{-};\forall k,i=2$$

The optimal solution of the economic benefit and eco-compensation of the model was obtained by calculating $t^{-}$ and $DIS_{jkmh}^{+}$ under different hydrological runoff conditions in the second stage and integrating the results of the upper limit sub-models and lower limit sub-models.

## 4. Results and Discussion

### 4.1. Analysis of the Optimization of the Water Resources Allocation Scheme

**Figure 2:**
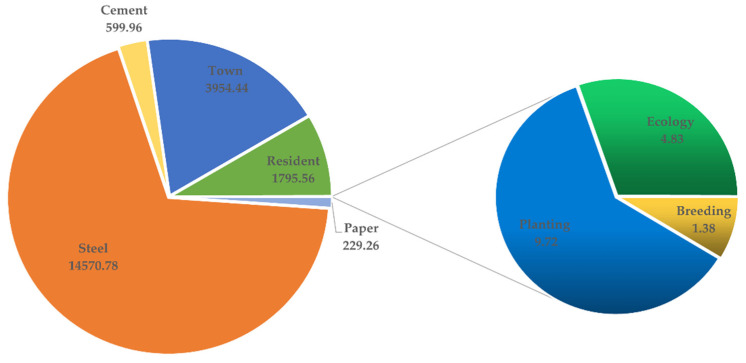
Adjustment of water resources utilization of different water sectors in Shanghang County in the first stage (×10^4^ t). (Where the Thermal power industry is not the main industry in Shanghang County, so the thermal power industry is not considered when allocating water resources, value of 0.).

Optimal allocation of water resources is an effective means for solving the uneven distribution of water resources in China, which is conducive to improving the utilization efficiency of the water resources [34,35]. Based on the ITS model data [27], the interval-fuzzy two-stage optimization method was used in this study to solve the optimization model of water resource allocation and eco-compensation mechanism for the Tingjiang River, and the value of the membership degree, $t^{\pm }$, was obtained as [0.08, 0.40]. The optimal water consumption of the water resources allocated to different departments of each administrative unit in the first stage was obtained based on the IFTS optimization method, as shown in Table 1. Taking Shanghang County of Fujian Province along the Tingjiang River as an example, the optimal allocation of water for consumption by different administrative units and different water consumption departments was analyzed based on the simulation results of the IFTS optimization model. Figure 2 shows the adjusted value of the utilization of water resources by different water-consuming departments in Shanghang County in the first stage. The industrial sector of Shanghang County is mainly composed of the papermaking industry, steel industry, and cement industry. To meet the industrial development planning of Shanghang County and the overall sustainable development requirements of the Tingjiang River, the optimization results from the IFTS optimization model predicted that Shanghang County in Fujian Province should allocate 229.26 × 10^4^ t of water resources to the paper industry, 14,570.78 × 10^4^ t to the steel industry, and 599.96 × 10^4^ t to the cement industry. In addition, 3954.44 × 10^4^ t of urban water resources in Shanghang County were allocated, and 1795.56 × 10^4^ t of water resources were allocated to residents in Shanghang County. The agricultural sector is mainly involved in two different industries, and 2.13 × 10^4^ t and 9.58 × 10^4^ t should be allocated for farming and planting, respectively. In order to ensure that the ecological environment of Shanghang County meets the relevant requirements, 3.22 × 10^4^ t of water resources were allocated to the ecological department of Shanghang County. Some of the parameters in Table 1 have a value of 0.00, indicating that the district or county did not consider that particular industry as the main industry or did not have the industry, and thus it had little influence on the water resources allocation. Therefore, the optimal allocation of water resources would not be carried out for this industry.

The upper limit sub-model was used for calculating the optimal solution of water resources allocation in the first stage. Since five different hydrological scenarios were set in this work, and the upper limit of water resources development and utilization and of the total water utilization were set as fuzzy variables, the model was further optimized to obtain a more scientific and stable solution. Based on the IFTS model, the $DIS_{h}^{\pm }$ penalty caused by the water quantity failing to meet the goal of the first stage was solved, i.e., the added value of water consumption caused by the failure of the optimal water quantity in the first stage, as shown in Table 2.

**Figure 3:**
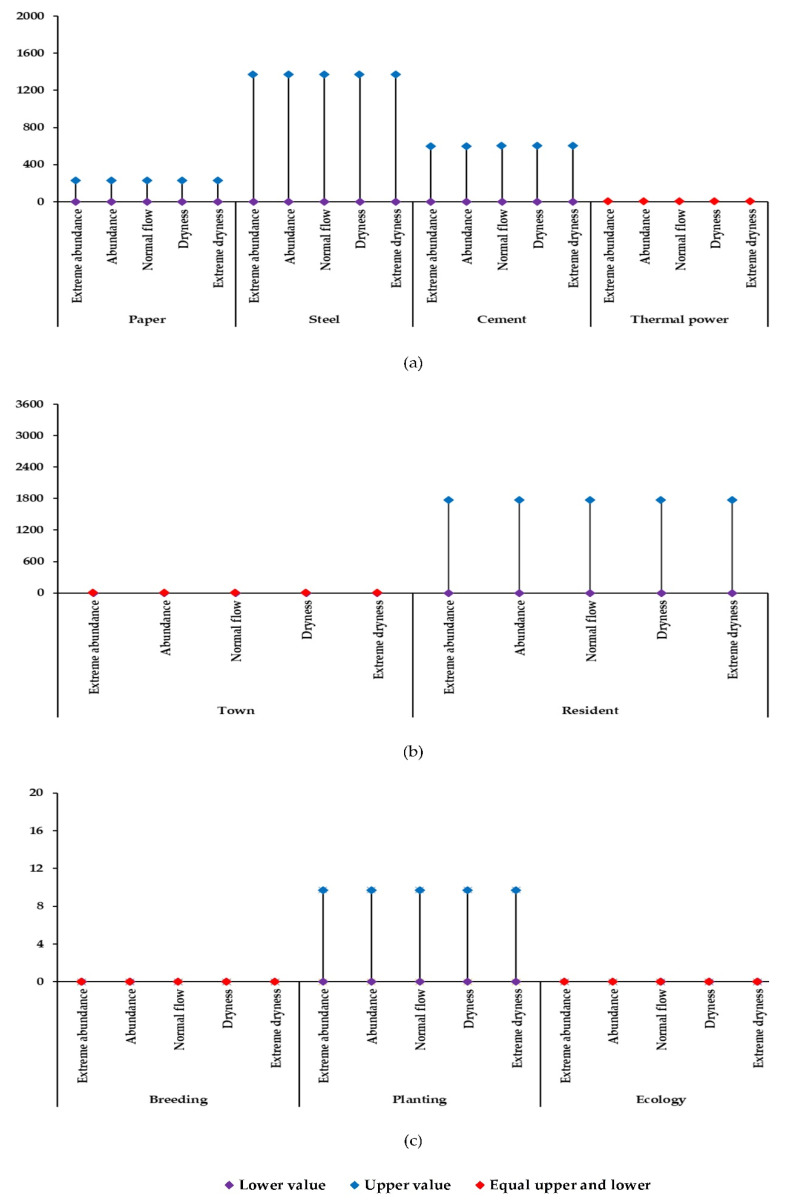
Different hydrological situations in different departments in the second stage of Shanghang water increment (×10^4^ t): (**a**) Industrial sectors; (**b**) Municipal sectors; (**c**) Agriculture and Ecology.

Figure 3 shows the increase in water usage by different departments in the second stage under different hydrological scenarios in Shanghang County. Industry in Shanghang County is mainly composed of papermaking, cement, and iron, and steel. Under different hydrological scenarios, different industries have different water increments in the second stage. Industry is an important driving force for economic development, and water affects industrial development [36]. To ensure regional economic benefits, it is necessary to ensure a basic industrial water supply. The paper industry requires an increased water supply of [0.00, 229.26] × 10^4^ t under the five hydrological scenarios of extreme dryness, dryness, normal flow, abundance, and extreme abundance. For the iron and steel industry, under five hydrological scenarios, Shanghang County should increase the water supply by [0.00, 1370.74] × 10^4^ t. Similarly, for the cement industry, the water supply needs to be increased to [0.00, 599.96] × 10^4^ t. In Shanghang County, the thermal power industry accounts for a relatively small proportion of the industry. Thus, it was considered to cause an increase in the water allocation requirement under the five hydrological scenarios.

Water plays an important role in human life [37,38]. In the allocation of water resources of the Tingjiang River to achieve social harmony and stability, it is necessary to ensure the supply of municipal water. Under different hydrological scenarios, Shanghang County needs to increase the supply of water resources by [0.00, 1767.72] × 10^4^ t. The development of the agriculture and breeding industry mainly depends on water supply [39,40]. To meet the water demand of basic agriculture and aquaculture development in Shanghang County, the water supply needs to be increased by [0.00, 1.38] × 10^4^ t in the extremely dry year, whereas, in the other four scenarios, there is no need to increase the water supply. For planting water in Shanghang County, under the five hydrological scenarios, it is necessary to increase the water supply to [0.00, 9.72] × 10^4^ t.

### 4.2. Analysis of the Optimization of Economic Benefits in the Tingjiang River Based on the IFTS Model

Based on the different hydrological scenarios and the IFTS model, the economic bene-fits of different administrative units in the upper and lower reaches of the Tingjiang River were calculated (Table 3 and Table 4). It was found that the economic benefits of some administrative units improved significantly, whereas some showed negative growth. Taking Liancheng County as an example, under the condition of an extremely dry year, due to a large amount of water shortage, industrial water distribution decreased, leading to the corresponding economic losses. This should be seen as the industry having the main positive factor affecting the economic benefits in the region. Compared to Liancheng County, the proportion of industry in other areas is small, and the fluctuation range of economic benefits was observed to be low, indicating that the policymakers increased the industrial water quota while ensuring the basic water demand of other sectors was met in order to create greater economic benefits within the availability of limited water resources.

According to the lower limit model calculated using the IFTS model, the range of variation of economic benefits in 14 districts and counties around the Tingjiang River was [−8.07%, 95.56%], in which the rate of decrease in the economic benefits was the largest in Dapu County of Guangdong Province, whereas the rate of increase was the largest in Wuping County of Fujian Province. Under the five hydrological scenarios, the total eco-nomic benefits were 3571.16 × 10^8^ CNY, 4046.44 × 10^8^ CNY, 3692.0^8^ × 10^8^ CNY, 3928.36 × 10^8^ CNY, and 3807.80 × 10^8^ CNY, respectively, and the probability level was 8%. At this time, the economic benefits were relatively low, but the decision-making risks were relatively small.

According to the simulation results of the upper limit sub-model of the IFTS model, the economic benefits of each region and county showed an upward trend after optimization of the model simulation, and the economic benefits of each region and county in-crease by [−2.12%, 8.33%]. Wuping County in Fujian province exhibited the lowest rate of economic benefit improvement, whereas Changting County in Fujian Province exhibited the largest. Under the five hydrological scenarios, the total economic benefits were 5749.98 × 10^8^ CNY, 5748.31 × 10^8^ CNY, 5749.63 × 10^8^ CNY, 5748.83 × 10^8^ CNY, and 5749.23 × 10^8^ CNY, respectively, and the possible level was 40%. However, pursuing the maximum economic benefits should bring great decision-making risks. The analysis of the optimization of economic benefits of the watershed shows that their reduction and improvement are in line with expectations, indicating that the IFTS model constructed in this work is reasonable and feasible to a certain extent.

### 4.3. Analysis of the Eco-Compensation Optimization of Tingjiang River Based on the IFTS Model

**Figure 4:**
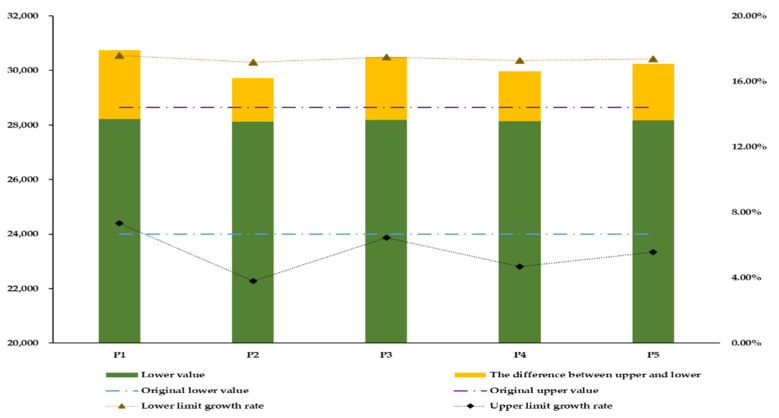
Optimization of the downstream region of Tingjiang River for the eco-compensation allocation of Guangdong Province (×10^4^ CNY).

As an important system to solve the problem of trans-regional water pollution, watershed eco-compensation can balance the interests of regional stakeholders and coordinate the development of the regional environment [41,42]. The eco-compensation quota of the upper reaches of the Tingjiang River was allocated according to the economic benefits of all administrative units in Guangdong Province. Table 5 and Table 6 list the eco-compensation ranges of Guangdong Province and Fujian Province obtained using the IFTS model under the five hydrological scenarios. The simulation results of the lower limit of the model show that the amount of eco-compensation paid by each downstream region increases. With the further development of the economy, the number of pollutants generated upstream will further increase, and the upstream pollution control capacity will face greater challenges. The increase in the eco-compensation can help to provide economic support for the upstream water environment management, improve the upstream pollution control capacity, and ensure that the upstream water quality meets the requirements of the water quality in the basin and the downstream water requirements. According to the lower limit IFTS sub-model, the range of variation in the eco-compensation quota in seven districts and counties in Guangdong Province was [12.13%, 23.44%]. The administrative unit with the smallest rate of increase of eco-compensation quota was Xingning County, whereas that with the largest rate of increase was Wuhua County. In addition, the total amount of eco-compensation decreased slowly with the decrease in the water quantity. According to the upper limit sub-model of the IFTS model, the eco-compensation quota changed within the range of [3.01%, 8.75%]. The administrative unit with the lowest rate of change of eco-compensation quota among the seven counties in Guangdong Province was Chenghai District, whereas that with the largest was Wuhua County. As can be seen from Figure 4, the lower limit of the total eco-compensation quota in Guangdong Province varied within the range of [17.16%, 17.58%], and the compensation quota varied by [28,116.40, 28,216.81] × 10^4^ CNY. The range of the upper limit was [3.81%, 7.33%], and the compensation amount varied by [30,738.60, 29,730.82] × 10^4^ CNY. The IFTS model fully considers five different hydrological scenarios and sets departmental and watershed water resources utilization thresholds as fuzzy variables, thus providing a broader decision space for decision-makers. Simultaneously, the simulation results show that the model is in line with the expectation of the eco-compensation scheme, and the feasibility of the model is verified from the perspective of eco-compensation.

### 4.4. Comparative Analysis of Fuzzy Optimization between the IFTS and ITS Models

Based on the ITS model that is based on the interval two-stage stochastic optimization method, the fuzzy optimization method was introduced in this study to construct the IFTS model. From the economic conditions of the basin and comparison of the optimized river basin eco-compensation amount, analysis of the effect of fuzzy uncertainty optimization scheme was performed.

**Figure 5:**
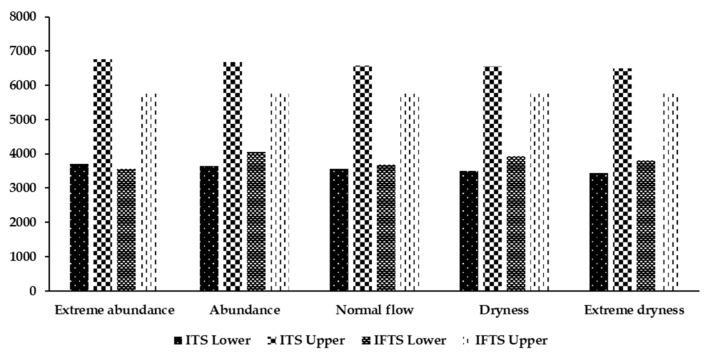
Comparison of the economic effect due to the interval value optimization using the ITS model and the IFTS model (×10^8^ CNY).

**Figure 6:**
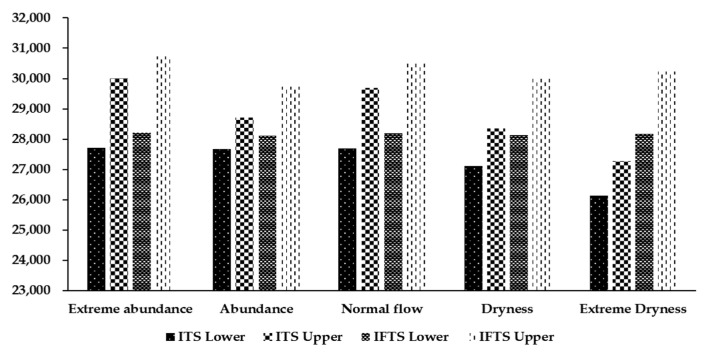
Comparison of the eco-compensation effect due to the interval value optimization model using the ITS model and the IFTS model (× 10^4^ CNY).

Figure 5 shows a comparison of the economic benefit optimization results obtained from the IFTS and the ITS models. After introducing the fuzzy optimization method, the economic benefit interval of the IFTS model increased by −28.54%, −44.09%, −31.49%, −40.37%, and −36.43%, respectively, under the five hydrological scenarios. The range of the interval values was reduced, the lower limit value of the ITS model was improved, and its upper limit value was reduced. Considering the uncertainty in the upper limit of water resources utilization by each department and the total basin under different hydrological scenarios, the stability and scientific nature of the decision-making system was enhanced, and a reasonable decision-making space was provided for the decision-makers. Figure 6 provides a comparison of the results obtained from the IFTS and the ITS optimization models for river basin eco-compensation. After introducing the fuzzy optimization method, the IFTS model was observed to increase the eco-compensation interval by 9.94%, 54.81%, 15.85%, 50.31%, and 82.90%, respectively, under the five hydrological scenarios, respectively. Basin eco-compensation has a wider range of choices, which provides more possibilities for coordinating economic interests and eco-compensation distribution in the upper and lower reaches of the basin, and the eco-compensation distribution is more scientific and reasonable.

## 5. Conclusions

Based on the ITS model, which is based on the interval two-stage stochastic optimization method, the fuzzy optimization method was introduced in this study to construct the IFTS model that incorporates the interval-fuzzy two-stage optimization method. The IFTS model can reduce the influence of the uncertain factors in the process of optimizing the water resource optimization and establishing an eco-compensation mechanism, narrow the range of the simulation results, and reduce the decision-making risks. In this work, the IFTS model was applied to optimize the allocation of water resources and eco-compensation mechanism for the Tingjiang River, which can maximize the overall economic benefit of this river while simultaneously achieving the objective of rational allocation of water resources and improvement of water resource utilization efficiency. The following are the key points corresponding to the IFTS model simulation results:

(1) In the second stage of the IFTS model simulation, the water supplement amount showed a downward trend compared to the ITS model simulation results, which effectively alleviated the water pressure of the different water-utilizing departments in each administrative unit around the Tingjiang River, coordinated the rights and interests of different water departments, and avoided the phenomenon of resource waste or limited development due to excessive water allocation.
(2) After introducing the fuzzy optimization method based on the ITS model, the upper limit of water resources utilization of different administrative units along the Tingjiang River and the upper limit of the total water resources utilization along the Tingjiang River were represented by fuzzy variables. By introducing the concept of a membership function to represent the possibility of a fuzzy variable value, the risk caused by the uncertain factors in the pursuit of economic maximization was effectively avoided, and the goal of economic benefit maximization under various hydrological scenarios (extreme dryness, dryness, normal flow, abundance, and extreme abundance) was realized.
(3) Compared to the simulation results of the ITS model, the upper limit of the total economic benefits of the Tingjiang River was significantly reduced, and the lower limit was significantly increased, indicating that the IFTS model shortens the decision-making space and improves the decision-making efficiency. In addition, using the IFTS model to simulate the range of total eco-compensation limit can effectively alleviate the upstream process of Fujian Province water environmental pollution problems and bear the economic pressures brought about by the water environment protection plan; thus, it improves the scientific nature and stability of the system.

## Figures and Tables

**Table 1:** Optimized allocation of water resources utilization of different water consumption sectors in each administrative unit in the upper and lower reaches of Tingjiang River based on the IFTS model in the first stage (× 10^4^ t).

Province	Section	Business	Liancheng	Shanghang	Wuping	Xinluo	Yongding	Pinghe	Changting
Fujian	Industry	Paper	324.93	229.26	621.66	701.21	533.77	569.29	264.55
		Steel	15,075.07	14,570.78	14,778.34	0.00	13,063.65	0.00	0.00
		Cement	0.00	599.96	0.00	437.93	1802.58	13,321.14	12,738.80
		Thermal power	0.00	0.00	0.00	14,260.86	0.00	1509.58	2264.37
	Municipal	Town	2875.63	3954.44	225.57	4472.20	1615.53	2790.91	3132.87
		Resident	2874.37	1795.56	5750.00	1277.80	4134.47	2959.09	2194.35
	Agriculture	Breeding	2.13	1.38	1.72	1.88	2.94	1.86	2.66
		Planting	9.58	9.72	11.07	27.04	25.20	25.22	5.25
	Ecology	Ecology	25.32	4.83	13.55	32.88	53.77	33.02	22.69
Guangdong			**Zijin**	**Dapu**	**Fengshun**	**Meixian**	**Wuhua**	**Xingning**	**Chenghai**
	Industry	Paper	259.92	224.70	213.25	426.00	320.07	469.39	215.52
		Steel	19,760.08	18,895.36	19,806.75	0.00	209.59	0.00	0.00
		Cement	0.00	899.94	0.00	19,335.35	19,490.35	19,324.17	222.07
		Thermal power	0.00	0.00	0.00	258.64	0.00	226.44	19,582.41
	Municipal	Town	4393.67	3322.66	3776.06	3507.54	3261.39	4064.78	3774.77
		Resident	3828.83	4152.34	4446.44	4714.96	4961.11	3410.22	3700.23
	Agriculture	Breeding	3.19	2.07	2.59	2.95	4.42	2.79	3.99
		Planting	14.37	14.58	16.61	13.76	90.48	37.84	7.88
	Ecology	Ecology	32.83	37.01	39.52	44.00	25.12	53.82	107.52

**Table 2:** Water additional configuration of IFTS model in phase 2 under different hydrological scenarios.

Number	Region	Section	Business	Penalty Amount in the Second Stage under Different Hydrological Scenarios (× 10^4^ t)				
				Extreme Abundance	Abundance	Normal Flow	Dryness	Extreme Dryness
1	Liancheng County	Industry	Paper	[0.00, 324.93]	[0.00, 324.93]	[0.00, 324.93]	[0.00, 324.93]	[0.00, 324.93]
			Steel	[0.00, 1275.07]	[0.00, 1275.07]	[0.00, 1275.07]	[0.00, 1275.07]	[0.00, 1275.07]
			Cement	[0.00, 0.00]	[0.00, 0.00]	[0.00, 0.00]	[0.00, 0.00]	[0.00, 0.00]
			Thermal power	[0.00, 0.00]	[0.00, 0.00]	[0.00, 0.00]	[0.00, 0.00]	[0.00, 0.00]
		Municipal	Town	[0.00, 0.00]	[0.00, 0.00]	[0.00, 0.00]	[0.00, 0.00]	[0.00, 0.00]
			Resident	[0.00, 2753.38]	[0.00, 2753.38]	[0.00, 2753.38]	[0.00, 2753.38]	[0.00, 2753.38]
		Agriculture	Breeding	[0.00, 0.00]	[0.00, 0.00]	[0.00, 0.00]	[0.00, 0.00]	[0.00, 0.00]
			Planting	[0.00, 9.58]	[0.00, 9.58]	[0.00, 9.58]	[0.00, 9.58]	[0.00, 9.58]
		Ecology	Ecology	[0.00, 0.00]	[0.00, 0.00]	[0.00, 0.00]	[0.00, 0.00]	[0.00, 0.00]
2	Shanghang County	Industry	Paper	[0.00, 229.26]	[0.00, 229.26]	[0.00, 229.26]	[0.00, 229.26]	[0.00, 229.26]
			Steel	[0.00, 1370.74]	[0.00, 1370.74]	[0.00, 1370.74]	[0.00, 1370.74]	[0.00, 1370.74]
			Cement	[0.00, 599.96]	[0.00, 599.96]	[0.00, 599.96]	[0.00, 599.96]	[0.00, 599.96]
			Thermal power	[0.00, 0.00]	[0.00, 0.00]	[0.00, 0.00]	[0.00, 0.00]	[0.00, 0.00]
		Municipal	Town	[0.00, 0.00]	[0.00, 0.00]	[0.00, 0.00]	[0.00, 0.00]	[0.00, 0.00]
			Resident	[0.00, 1767.72]	[0.00, 1767.72]	[0.00, 1767.72]	[0.00, 1767.72]	[0.00, 1767.72]
		Agriculture	Breeding	[0.00, 0.00]	[0.00, 0.00]	[0.00, 0.00]	[0.00, 0.00]	[0.00, 0.00]
			Planting	[0.00, 9.72]	[0.00, 9.72]	[0.00, 9.72]	[0.00, 9.72]	[0.00, 9.72]
		Ecology	Ecology	[0.00, 0.00]	[0.00, 0.00]	[0.00, 0.00]	[0.00, 0.00]	[0.00, 0.00]
3	Wuping County	Industry	Paper	[0.00, 621.66]	[0.00, 621.66]	[0.00, 621.66]	[0.00, 621.66]	[0.00, 621.66]
			Steel	[0.00, 978.34]	[0.00, 978.34]	[0.00, 978.34]	[0.00, 978.34]	[0.00, 978.34]
			Cement	[0.00, 0.00]	[0.00, 0.00]	[0.00, 0.00]	[0.00, 0.00]	[0.00, 0.00]
			Thermal power	[0.00, 0.00]	[0.00, 0.00]	[0.00, 0.00]	[0.00, 0.00]	[0.00, 0.00]
		Municipal	Town	[0.00, 225.57]	[0.00, 225.57]	[0.00, 225.57]	[0.00, 225.57]	[0.00, 225.57]
			Resident	[0.00, 4086.50]	[0.00, 4086.50]	[0.00, 4086.50]	[0.00, 4086.50]	[0.00, 4086.50]
		Agriculture	Breeding	[0.00, 0.00]	[0.00, 0.00]	[0.00, 0.00]	[0.00, 0.00]	[0.00, 0.00]
			Planting	[0.00, 11.07]	[0.00, 11.07]	[0.00, 11.07]	[0.00, 11.07]	[0.00, 11.07]
		Ecology	Ecology	[0.00, 0.00]	[0.00, 0.00]	[0.00, 0.00]	[0.00, 0.00]	[0.00, 0.00]
4	Xinluo District	Industry	Paper	[0.00, 701.21]	[0.00, 701.21]	[0.00, 701.21]	[0.00, 701.21]	[0.00, 701.21]
			Steel	[0.00, 0.00]	[0.00, 0.00]	[0.00, 0.00]	[0.00, 0.00]	[0.00, 0.00]
			Cement	[0.00, 437.93]	[0.00, 437.93]	[0.00, 437.93]	[0.00, 437.93]	[0.00, 437.93]
			Thermal power	[0.00, 460.86]	[0.00, 460.86]	[0.00, 460.86]	[0.00, 460.86]	[0.00, 460.86]
		Municipal	Town	[0.00, 1150.00]	[0.00, 1150.00]	[0.00, 0.00]	[0.00, 1150.00]	[0.00, 0.00]
			Resident	[0.00, 0.00]	[0.00, 0.00]	[0.00, 1150.00]	[0.00, 0.00]	[0.00, 1150.00]
		Agriculture	Breeding	[0.00, 0.00]	[0.00, 0.00]	[0.00, 1.88]	[0.00, 0.00]	[0.00, 0.00]
			Planting	[0.00, 0.00]	[0.00, 0.00]	[0.00, 0.00]	[0.00, 0.00]	[0.00, 0.00]
		Ecology	Ecology	[0.00, 0.00]	[0.00, 0.00]	[0.00, 0.00]	[0.00, 0.00]	[0.00, 0.00]
5	Yongding District	Industry	Paper	[0.00, 533.77]	[0.00, 533.77]	[0.00, 533.77]	[0.00, 533.77]	[0.00, 533.77]
			Steel	[0.00, 0.00]	[0.00, 0.00]	[0.00, 0.00]	[0.00, 0.00]	[0.00, 0.00]
			Cement	[0.00, 1066.23]	[0.00, 1066.23]	[0.00, 1066.23]	[0.00, 1066.23]	[0.00, 1066.23]
			Thermal power	[0.00, 0.00]	[0.00, 0.00]	[0.00, 0.00]	[0.00, 0.00]	[0.00, 0.00]
		Municipal	Town	[0.00, 0.00]	[0.00, 0.00]	[0.00, 0.00]	[0.00, 0.00]	[0.00, 0.00]
			Resident	[0.00, 1778.84]	[0.00, 1778.84]	[0.00, 1778.84]	[0.00, 1778.84]	[0.00, 1778.84]
		Agriculture	Breeding	[0.00, 2.94]	[0.00, 2.94]	[0.00, 2.94]	[0.00, 2.94]	[0.00, 2.94]
			Planting	[0.00, 0.44]	[0.00, 0.44]	[0.00, 0.44]	[0.00, 0.44]	[0.00, 0.44]
		Ecology	Ecology	[0.00, 0.00]	[0.00, 0.00]	[0.00, 0.00]	[0.00, 0.00]	[0.00, 0.00]
6	Pinghe County	Industry	Paper	[0.00, 411.21]	[0.00, 490.37]	[0.00, 490.37]	[0.00, 490.37]	[0.00, 411.21]
			Steel	[0.00, 0.00]	[0.00, 0.00]	[0.00, 0.00]	[0.00, 0.00]	[0.00, 0.00]
			Cement	[0.00, 0.00]	[0.00, 0.00]	[0.00, 2890.12]	[0.00, 2764.73]	[0.00, 2597.40]
			Thermal power	[0.00, 1188.79]	[0.00, 1109.63]	[0.00, 1109.63]	[0.00, 1109.63]	[0.00, 1188.79]
		Municipal	Town	[0.00, 0.00]	[0.00, 1150.00]	[0.00, 1150.00]	[0.00, 1150.00]	[0.00, 0.00]
			Resident	[0.00, 1150.00]	[0.00, 0.00]	[0.00, 0.00]	[0.00, 0.00]	[0.00, 1150.00]
		Agriculture	Breeding	[0.00, 0.00]	[0.00, 0.00]	[0.00, 0.00]	[0.00, 0.00]	[0.00, 0.00]
			Planting	[0.00, 0.00]	[0.00, 0.00]	[0.00, 0.00]	[0.00, 0.00]	[0.00, 0.00]
		Ecology	Ecology	[0.00, 0.00]	[0.00, 0.00]	[0.00, 0.00]	[0.00, 0.00]	[0.00, 0.00]
7	Changting County	Industry	Paper	[0.00, 167.16]	[0.00, 167.16]	[0.00, 167.16]	[0.00, 167.16]	[0.00, 167.16]
			Steel	[0.00, 0.00]	[0.00, 0.00]	[0.00, 0.00]	[0.00, 0.00]	[0.00, 0.00]
			Cement	[0.00, 0.00]	[0.00, 0.00]	[0.00, 0.00]	[0.00, 0.00]	[0.00, 0.00]
			Thermal power	[0.00, 1300.57]	[0.00, 1300.57]	[0.00, 1300.57]	[0.00, 1300.57]	[0.00, 1250.52]
		Municipal	Town	[0.00, 0.00]	[0.00, 0.00]	[0.00, 0.00]	[0.00, 0.00]	[0.00, 727.22]
			Resident	[0.00, 727.22]	[0.00, 727.22]	[0.00, 727.22]	[0.00, 727.22]	[0.00, 0.00]
		Agriculture	Breeding	[0.00, 0.00]	[0.00, 0.00]	[0.00, 0.00]	[0.00, 0.00]	[0.00, 0.00]
			Planting	[0.00, 0.00]	[0.00, 0.00]	[0.00, 0.00]	[0.00, 0.00]	[0.00, 0.00]
		Ecology	Ecology	[0.00, 0.00]	[0.00, 0.00]	[0.00, 0.00]	[0.00, 0.00]	[0.00, 0.00]
8	Zijin County	Industry	Paper	[0.00, 259.92]	[18.30, 259.92]	[21.35, 259.92]	[21.35, 259.92]	[259.92, 259.92]
			Steel	[0.00, 1929.52]	[0.00, 1929.52]	[0.00, 1929.52]	[0.00, 1929.52]	[0.00, 1929.52]
			Cement	[0.00, 0.00]	[0.00, 0.00]	[0.00, 0.00]	[0.00, 0.00]	[0.00, 0.00]
			Thermal power	[0.00, 0.00]	[0.00, 0.00]	[0.00, 0.00]	[0.00, 0.00]	[0.00, 0.00]
		Municipal	Town	[0.00, 747.50]	[0.00, 747.50]	[0.00, 747.50]	[0.00, 747.50]	[0.00, 747.50]
			Resident	[0.00, 3828.83]	[0.00, 3828.83]	[0.00, 3828.83]	[0.00, 3828.83]	[0.00, 3828.83]
		Agriculture	Breeding	[0.00, 3.19]	[0.00, 3.19]	[0.00, 3.19]	[0.00, 3.19]	[0.00, 3.19]
			Planting	[0.00, 14.37]	[0.00, 14.37]	[0.00, 14.37]	[0.00, 14.37]	[0.00, 14.37]
		Ecology	Ecology	[0.00, 0.00]	[0.00, 0.00]	[0.00, 0.00]	[0.00, 0.00]	[0.00, 0.00]
9	Dapu County	Industry	Paper	[0.00, 224.70]	[0.00, 224.70]	[0.00, 224.70]	[0.00, 224.70]	[0.00, 224.70]
			Steel	[0.00, 1855.30]	[0.00, 1855.30]	[0.00, 1855.30]	[0.00, 1855.30]	[0.00, 1855.30]
			Cement	[0.00, 0.00]	[0.00, 0.00]	[0.00, 0.00]	[0.00, 0.00]	[0.00, 0.00]
			Thermal power	[0.00, 0.00]	[0.00, 0.00]	[0.00, 0.00]	[0.00, 0.00]	[0.00, 0.00]
		Municipal	Town	[0.00, 1495.00]	[0.00, 1495.00]	[0.00, 1495.00]	[0.00, 1495.00]	[0.00, 1495.00]
			Resident	[0.00, 0.00]	[0.00, 0.00]	[0.00, 0.00]	[0.00, 0.00]	[0.00, 0.00]
		Agriculture	Breeding	[0.00, 0.00]	[0.00, 0.00]	[0.00, 0.00]	[0.00, 0.00]	[0.00, 0.00]
			Planting	[0.00, 0.00]	[0.00, 0.00]	[0.00, 0.00]	[0.00, 0.00]	[0.00, 0.00]
		Ecology	Ecology	[0.00, 0.00]	[0.00, 0.00]	[0.00, 0.00]	[0.00, 0.00]	[0.00, 0.00]
10	Fengshun County	Industry	Paper	[0.00, 213.25]	[0.00, 213.25]	[0.00, 213.25]	[0.00, 213.25]	[0.00, 213.25]
			Steel	[0.00, 1866.75]	[0.00, 1866.75]	[0.00, 1866.75]	[0.00, 1866.75]	[0.00, 1866.75]
			Cement	[0.00, 0.00]	[0.00, 0.00]	[0.00, 0.00]	[0.00, 0.00]	[0.00, 0.00]
			Thermal power	[0.00, 0.00]	[0.00, 0.00]	[0.00, 0.00]	[0.00, 0.00]	[0.00, 0.00]
		Municipal	Town	[0.00, 747.50]	[0.00, 747.50]	[0.00, 747.50]	[0.00, 747.50]	[0.00, 747.50]
			Resident	[0.00, 4053.46]	[0.00, 4053.46]	[0.00, 4053.46]	[0.00, 4053.46]	[0.00, 4053.46]
		Agriculture	Breeding	[0.00, 2.59]	[0.00, 2.59]	[0.00, 2.59]	[0.00, 2.59]	[0.00, 2.59]
			Planting	[0.00, 16.61]	[0.00, 16.61]	[0.00, 16.61]	[0.00, 16.61]	[0.00, 16.61]
		Ecology	Ecology	[0.00, 0.00]	[0.00, 0.00]	[0.00, 0.00]	[0.00, 0.00]	[0.00, 0.00]
11	Meixian District	Industry	Paper	[0.00, 426.00]	[0.00, 426.00]	[0.00, 426.00]	[0.00, 426.00]	[0.00, 426.00]
			Steel	[0.00, 0.00]	[0.00, 0.00]	[0.00, 0.00]	[0.00, 0.00]	[0.00, 0.00]
			Cement	[0.00, 1395.35]	[0.00, 1395.35]	[0.00, 1395.35]	[0.00, 1395.35]	[0.00, 1395.35]
			Thermal power	[258.64, 258.64]	[258.64, 258.64]	[0.00, 258.64]	[0.00, 258.64]	[0.00, 258.64]
		Municipal	Town	[747.50, 1569.74]	[747.50, 1569.74]	[1006.14, 1569.74]	[1006.14, 1569.74]	[1006.14, 1569.74]
			Resident	[0.00, 672.76]	[0.00, 672.76]	[0.00, 672.76]	[0.00, 672.76]	[0.00, 672.76]
		Agriculture	Breeding	[0.00, 0.00]	[0.00, 0.00]	[0.00, 0.00]	[0.00, 0.00]	[0.00, 0.00]
			Planting	[0.00, 0.00]	[0.00, 0.00]	[0.00, 0.00]	[0.00, 0.00]	[0.00, 0.00]
		Ecology	Ecology	[0.00, 0.00]	[0.00, 0.00]	[0.00, 0.00]	[0.00, 0.00]	[0.00, 0.00]
12	Wuhua County	Industry	Paper	[0.00, 320.07]	[0.00, 320.07]	[0.00, 320.07]	[0.00, 320.07]	[0.00, 320.07]
			Steel	[0.00, 209.59]	[0.00, 209.59]	[0.00, 209.59]	[0.00, 209.59]	[0.00, 209.59]
			Cement	[0.00, 1550.35]	[0.00, 1550.35]	[0.00, 1550.35]	[0.00, 1550.35]	[0.00, 1550.35]
			Thermal power	[0.00, 0.00]	[0.00, 0.00]	[0.00, 0.00]	[0.00, 0.00]	[0.00, 0.00]
		Municipal	Town	[416.43, 747.50]	[416.43, 747.50]	[416.43, 747.50]	[416.43, 747.50]	[416.43, 747.50]
			Resident	[0.00, 1826.07]	[0.00, 1826.07]	[0.00, 1826.07]	[0.00, 1826.07]	[0.00, 1826.07]
		Agriculture	Breeding	[0.00, 0.00]	[0.00, 0.00]	[0.00, 0.00]	[0.00, 0.00]	[0.00, 0.00]
			Planting	[0.00, 0.00]	[0.00, 0.00]	[0.00, 0.00]	[0.00, 0.00]	[0.00, 0.00]
		Ecology	Ecology	[0.00, 0.00]	[0.00, 0.00]	[0.00, 0.00]	[0.00, 0.00]	[0.00, 0.00]
13	Xingning County	Industry	Paper	[0.00, 469.39]	[0.00, 469.39]	[0.00, 469.39]	[0.00, 469.39]	[0.00, 469.39]
			Steel	[0.00, 0.00]	[0.00, 0.00]	[0.00, 0.00]	[0.00, 0.00]	[0.00, 0.00]
			Cement	[0.00, 1384.17]	[0.00, 1384.17]	[0.00, 1384.17]	[0.00, 1384.17]	[0.00, 1384.17]
			Thermal power	[226.44, 226.44]	[226.44, 226.44]	[226.44, 226.44]	[226.44, 226.44]	[226.44, 226.44]
		Municipal	Town	[0.00, 4064.78]	[0.00, 4064.78]	[0.00, 4064.78]	[4064.78, 4064.78]	[4064.78, 4064.78]
			Resident	[0.00, 1495.00]	[0.00, 1495.00]	[0.00, 0.00]	[0.00, 1495.00]	[0.00, 1495.00]
		Agriculture	Breeding	[0.00, 0.00]	[0.00, 0.00]	[0.00, 1495.00]	[0.00, 0.00]	[0.00, 0.00]
			Planting	[0.00, 0.00]	[0.00, 0.00]	[0.00, 0.00]	[0.00, 0.00]	[0.00, 0.00]
		Ecology	Ecology	[0.00, 0.00]	[0.00, 0.00]	[0.00, 0.00]	[0.00, 0.00]	[0.00, 0.00]
14	Chenghai District	Industry	Paper	[0.00, 215.52]	[0.00, 215.52]	[0.00, 215.52]	[0.00, 215.52]	[0.00, 215.52]
			Steel	[0.00, 0.00]	[0.00, 0.00]	[0.00, 0.00]	[0.00, 0.00]	[0.00, 0.00]
			Cement	[0.00, 222.07]	[0.00, 222.07]	[0.00, 222.07]	[0.00, 222.07]	[0.00, 222.07]
			Thermal power	[0.00, 1624.41]	[0.00, 1624.41]	[0.00, 1624.41]	[0.00, 1624.41]	[0.00, 1624.41]
		Municipal	Town	[0.00, 1494.07]	[0.00, 0.00]	[0.00, 1494.07]	[0.00, 0.00]	[0.00, 0.00]
			Resident	[0.00, 0.93]	[0.00, 1495.00]	[0.00, 0.93]	[0.00, 1495.00]	[0.00, 1495.00]
		Agriculture	Breeding	[0.00, 0.00]	[0.00, 0.00]	[0.00, 0.00]	[0.00, 0.00]	[0.00, 0.00]
			Planting	[0.00, 0.00]	[0.00, 0.00]	[0.00, 0.00]	[0.00, 0.00]	[0.00, 0.00]
		Ecology	Ecology	[0.00, 0.00]	[0.00, 0.00]	[0.00, 0.00]	[0.00, 0.00]	[0.00, 0.00]

**Table 3:** Economic benefits of each administrative unit simulated by the IFTS model under different hydrological scenarios (lower limit).

Number	Region	Lower Limit (×10^8^ CNY)	The Lower Limit of the IFTS Model Is the Benefit of Each Region in Each Hydrological Scenario (×10^8^ CNY)					Change (%)
			*p* (1)	*p* (2)	*p* (3)	*p* (4)	*p* (5)	
1	Liancheng	165.47	197.57	214.05	201.69	209.93	205.81	[19.40, 29.36]
2	Shanghang	313.07	370.44	377.48	372.20	375.72	373.96	[18.32, 20.57]
3	Wuping	111.80	181.48	218.64	190.77	209.35	200.06	[62.33, 95.56]
4	Xinluo	767.60	905.63	945.21	915.43	935.31	925.29	[17.98, 23.14]
5	Yongding	184.43	239.50	270.36	247.21	262.64	254.93	[29.86, 46.59]
6	Pinghe	219.29	241.65	279.30	253.27	270.62	256.07	[10.20, 27.37]
7	Changting	207.96	271.59	280.50	273.82	278.27	279.59	[30.60, 34.88]
8	Zijin	89.85	88.98	125.81	98.19	116.60	107.39	[−0.97, 40.02]
9	Dapu	61.37	56.42	86.07	63.83	78.66	71.25	[−8.07, 40.25]
10	Fengshun	67.29	65.47	96.02	73.11	88.39	80.75	[−2.70, 42.70]
11	Meixian	164.19	165.03	227.41	180.63	211.82	196.22	[0.51, 38.50]
12	Wuhua	127.47	129.45	175.39	140.94	163.91	152.42	[1.55, 37.59]
13	Xingning	149.59	143.34	212.88	160.71	195.49	178.11	[−4.18, 42.31]
14	Chenghai	439.93	514.61	537.32	520.29	531.64	525.96	[16.98, 22.14]
Total		3069.32	3571.16	4046.44	3692.08	3928.36	3807.80	[16.35, 31.84]

**Table 4:** Economic benefits of each administrative unit simulated by the IFTS model under different hydrological scenarios (upper limit).

Number	Region	Upper Limit (×10^8^ CNY)	The Upper Limit of the IFTS Model Is the Benefit of Each Region in Each Hydrological Scenario (×10^8^ CNY)					Change (%)
			*p* (1)	*p* (2)	*p* (3)	*p* (4)	*p* (5)	
1	Liancheng	315.99	309.02	309.02	309.02	309.02	309.02	[−2.11, −2.11]
2	Shanghang	504.58	496.45	496.05	496.35	496.15	496.25	[−1.69, −1.61]
3	Wuping	337.78	330.78	330.61	330.74	330.65	330.70	[−2.12, −2.07]
4	Xinluo	1160.54	1157.88	1157.88	1157.88	1157.88	1157.88	[−0.23, −0.23]
5	Yongding	390.41	382.97	382.97	382.97	382.97	382.97	[−1.91, −1.91]
6	Pinghe	376.92	370.16	370.16	370.16	370.16	370.16	[−1.79, −1.79]
7	Changting	333.62	361.41	361.41	361.41	361.41	361.41	[8.33, 8.33]
8	Zijin	278.99	280.11	279.88	280.05	279.94	279.99	[0.32, 0.40]
9	Dapu	198.00	198.09	198.09	198.09	198.09	198.09	[0.05, 0.05]
10	Fengshun	236.99	238.11	237.88	238.05	237.94	238.00	[0.38, 0.47]
11	Meixian	341.22	342.25	341.86	342.22	342.07	342.14	[0.19, 0.30]
12	Wuhua	268.50	269.62	269.39	269.56	269.45	269.51	[0.33, 0.42]
13	Xingning	329.36	329.27	329.24	329.26	329.25	329.26	[−0.04, −0.03]
14	Chenghai	683.90	683.85	683.85	683.85	683.85	683.85	[−0.01, −0.01]
Total		5756.79	5749.98	5748.31	5749.63	5748.83	5749.23	[−0.12, −0.15]

**Table 5:** Eco-compensation allocation of the downstream administrative units under different hydrological scenarios based on the IFTS model (lower limit).

Number	Region	Lower Limit (×10^4^ CNY)	Allocation of Eco-Compensation in Different Regions and Counties under Different Hydrological Scenarios (×10^4^ CNY)					Change (%)
			*p* (1)	*p* (2)	*p* (3)	*p* (4)	*p* (5)	
8	Zijin	3366.49	4071.68	4050.07	4066.27	4055.47	4060.85	[20.31, 20.95]
9	Dapu	3534.36	3974.36	3974.36	3974.36	3974.36	3974.36	[12.45, 12.45]
10	Fengshun	3375.64	4071.91	4050.31	4066.51	4055.71	4061.11	[19.99, 20.63]
11	Meixian	3333.33	4067.81	4038.74	4060.55	4060.01	4053.29	[21.16, 22.03]
12	Wuhua	3307.43	4082.84	4061.25	4077.46	4066.65	4072.05	[22.79, 23.44]
13	Xingning	3538.70	3974.55	3968.01	3972.91	3969.64	3971.28	[12.13, 12.32]
14	Chenghai	3541.77	3973.66	3973.66	3973.66	3973.66	3973.66	[12.19, 12.19]
Total		23,997.72	28,216.81	28,116.40	28,191.71	28,141.50	28,166.60	[17.16, 17.58]

**Table 6:** Eco-compensation allocation of the downstream administrative units under different hydrological scenarios based on the IFTS model (upper limit).

Number	Region	Upper Limit (×10^4^ CNY)	Allocation of Eco-Compensation in Different Regions and Counties under Different Hydrological Scenarios (×10^4^ CNY)					Change (%)
			*p* (1)	*p* (2)	*p* (3)	*p* (4)	*p* (5)	
8	Zijin	4087.21	4415.83	4223.09	4367.65	4271.27	4319.46	[3.32, 8.04]
9	Dapu	4087.69	4335.63	4221.47	4307.09	4250.01	4278.55	[3.27, 6.07]
10	Fengshun	4088.42	4414.23	4217.75	4365.11	4266.87	4315.99	[3.16, 7.97]
11	Meixian	4088.70	4443.05	4305.02	4408.55	4339.53	4374.04	[5.29, 8.67]
12	Wuhua	4097.17	4455.54	4317.50	4421.03	4352.01	4386.52	[5.38, 8.75]
13	Xingning	4093.58	4339.46	4225.29	4310.92	4253.83	4282.37	[3.22, 6.01]
14	Chenghai	4097.18	4334.86	4220.70	4306.32	4249.25	4277.78	[3.01, 5.80]
Total		28,639.95	30,738.60	29,730.82	30,486.65	29,982.77	30,234.71	[3.81, 7.33]

## Data Availability

The data presented in this study are available contained within the article.

## Abbreviations

$f_{1}^{\pm }$	Water economic benefit, 10^4^ CNY.
$f_{2}^{\pm }$	Water consumption cost, 10^4^ CNY.
$f_{3}^{\pm }$	Cost of water environment management, 10^4^ CNY.
$f_{4}^{\pm }$	Eco-compensation quota, 10^4^ CNY.
$f_{5}^{\pm }$	The second stage is to optimize the penalty value.
$f^{\prime }$	The lowest economic benefit of the basin, 10^4^ CNY.
$f^{\prime \prime }$	The highest economic benefit of the basin, 10^4^ CNY.
j	Administrative units (14 districts and counties).
k	Major water consumption sectors (k = 1, 2, 3, 4 denote Industry, Municipal, Agriculture, Ecology).
m	Different industry categories within each sector
i	Watershed partition (*i* = 1 denotes regional scope of upstream Fujian Province; *i* = 2 denotes regional scope of downstream Guangdong Province).
h	Hydrological situation (h = 1, 2, 3, 4, 5 denote extreme abundance, abundance, normal flow, dryness, and extreme dryness, and the probabilities are respectively 0.1, 0.3, 0.15, 0.25, and 0.2).
$NB_{jkm}^{\pm }$	Output value per unit scale, 10^4^ CNY/10^4^ t.
$ISOPT_{jkm}$	Optimal solution of water consumption in the first stage.
$WR_{jk}^{\pm }$	The unit price with water, 10^4^ CNY/10^4^ t.
$\delta_{jkm}^{\pm }$	Comprehensive pollution production coefficient, 10^4^ g/10^4^ t.
$STC_{jkm}^{\pm }$	Pollution control cost, 10^4^ CNY/10^4^ t.
$WRD_{j}^{\pm }$	Downstream water price, 10^4^ CNY/10^4^ t.
η	The downstream uses the proportion of incoming water from the upstream.
λ	Eco-compensation determination coefficient (the water quality is better than the III standard, λ = 1; the water quality is inferior to class V, λ = –1; in other cases, λ = 0).
P	Hydrological scenario probability.
$PNB_{h}^{\pm }$	The water supply cannot meet the loss caused by the original water supply, 10^4^ CNY/10^4^ t.
$DIS_{h}^{\pm }$	Lack of water, 10^4^ t.
$IWUL_{jk}^{\pm }$	The maximum water resources utilization stipulated by different regions and departments, 10^4^ t.
$RWUL_{j}^{\pm }$	The maximum utilization of water resources in different regions, 10^4^ t.
$ECS_{j}^{\pm }$	The ecological area range of different regions in the watershed, 10^4^ t.
$TAWR^{\pm }$	The maximum utilization of water resources in the basin, 10^4^ t.
r	Different pollutants.
$\xi_{jkmr}^{\pm }$	Pollutants producing coefficient, 10^4^ g/10^4^ t.
$\gamma_{jkmr}^{\pm }$	Pollutant removal rate.
$AEP_{jr}^{\pm }$	Maximum allowable discharge of pollutants, 10^4^ t.
$DSL_{ik}^{\pm }$	Minimum regional development requirements, 10^4^ CNY.
